# Hydrogen Peroxide and Abscisic Acid Mediate Salicylic Acid-Induced Freezing Tolerance in Wheat

**DOI:** 10.3389/fpls.2018.01137

**Published:** 2018-08-03

**Authors:** Weiling Wang, Xiao Wang, Mei Huang, Jian Cai, Qin Zhou, Tingbo Dai, Weixing Cao, Dong Jiang

**Affiliations:** National Technique Innovation Center for Regional Wheat Production, Key Laboratory of Crop Physiology and Ecology in Southern China, Ministry of Agriculture, National Engineering and Technology Center for Information Agriculture, Nanjing Agricultural University, Nanjing, China

**Keywords:** endogenous signal, freezing tolerance, inhibitor, salicylic acid, wheat

## Abstract

Salicylic acid (SA) can induce plant resistance to biotic and abiotic stresses through cross talk with other signaling molecules, whereas the interaction between hydrogen peroxide (H_2_O_2_) and abscisic acid (ABA) in response to SA signal is far from clear. Here, we focused on the roles and interactions of H_2_O_2_ and ABA in SA-induced freezing tolerance in wheat plants. Exogenous SA pretreatment significantly induced freezing tolerance of wheat via maintaining relatively higher dark-adapted maximum photosystem II quantum yield, electron transport rates, less cell membrane damage. Exogenous SA induced the accumulation of endogenous H_2_O_2_ and ABA. Endogenous H_2_O_2_ accumulation in the apoplast was triggered by both cell wall peroxidase and membrane-linked NADPH oxidase. The pharmacological study indicated that pretreatment with dimethylthiourea (H_2_O_2_ scavenger) completely abolished SA-induced freezing tolerance and ABA synthesis, while pretreatment with fluridone (ABA biosynthesis inhibitor) reduced H_2_O_2_ accumulation by inhibiting NADPH oxidase encoding genes expression and partially counteracted SA-induced freezing tolerance. These findings demonstrate that endogenous H_2_O_2_ and ABA signaling may form a positive feedback loop to mediate SA-induced freezing tolerance in wheat.

## Introduction

As one of the major food crops, wheat (*Triticum aestivum* L.) frequently suffers from freezing stress, especially during jointing stage. Freezing affects the development of the young spikelet, hibits plant growth, and grain yield formation (Kosova et al., 2013). To overcome freezing stress constraint, plants have developed highly sophisticated and intricate defense mechanisms to enhance stresses tolerance, which are largely dependent on the delicate signaling cascades network, activation of the reactive oxygen scavenging systems, accumulation of the compatible solutes or osmosis protectants, expression of the cold-responsive genes (Ruellan et al., 2009).

Salicylic acid (SA) is a naturally occurring phenolic compound and key signaling molecule that plays an essential role in the regulation of diverse physiological processes in plants (An and Mou, 2011; Yang et al., 2013). SA has been widely investigated for its crucial role in mediating plant responses to pathogen infection, such as inducing host cell death and systemic acquired resistance (SAR) (Yoshimoto et al., 2009; Kalachova et al., 2013). Apart from this role, SA has received much attention due to its role in plants response to abiotic stresses (e.g., drought, extreme temperature, heavy metal, and salinity) (Miura and Tada, 2014; Guo et al., 2016). Previous studies have shown that low temperature could induce endogenous SA accumulation in plants (Scott et al., 2004; Dong et al., 2014). Pretreated with SA biosynthesis inhibitors significantly down-regulated the expression of cold-responsive genes in cucumber (Dong et al., 2014), decreased the capacity of antioxidant in watermelon (Fei et al., 2016), resulting in a reduction in cold tolerance. These findings demonstrated that endogenous SA plays an important role in plant response to low temperature stress. Exogenous SA can enhance plant tolerance to cold stress, through increasing antioxidant capacity and polyamine content in maize (Janda et al., 1999; Nemeth et al., 2002), and apoplastic antifreeze protein accumulation in wheat (Tasgin et al., 2003). However, the mechanism of SA-induced tolerance mediated by signaling molecules in response to freezing stress remain to be investigated.

Hydrogen peroxide (H_2_O_2_), is considered as a second messenger in phytohormone signalings and is involved in the regulation of plant responses to abiotic stresses (Xia et al., 2009; Maruta et al., 2012). It appears that SA-induced H_2_O_2_ is necessary for SAR during pathogen infection (Chen et al., 1993). Moreover, the self-amplifying feedback loop between SA and H_2_O_2_ indicated that there is a synergistic interaction to mediate plant cell death in *atg* (autophagy) mutant (Yoshimoto et al., 2009) or during pathogen infection (Vlot et al., 2009). Exogenous SA-induced H_2_O_2_ can be produced through several pathways, including increasing the activity of cell wall Prx (Mori et al., 2001) or membrane-linked NADPH oxidase (Agarwala et al., 2005), inhibiting the activity of CAT or APX (Kang et al., 2003). However, the pathway of SA-induced endogenous H_2_O_2_ production remains largely elusive in wheat.

Abscisic acid (ABA) is a central component in plants response to freezing stress (Lang et al., 1994; Akter et al., 2014). Both ABA and SA signal transduction pathways are activated by cold, which implies their involvement in cold stress responses in rice (Zhao et al., 2015). Moreover, Pal et al. (2011) found that SA participates in ABA-induced cold acclimation in maize. Furthermore, ABA also plays a predominant role in exogenous SA-induced tolerance to salt stress in tomato (Horvath et al., 2015) and cadmium toxicity in wheat (Shakirova et al., 2016). These findings suggest that endogenous ABA may be involved in SA-induced resistance to freezing stress in wheat plants.

H_2_O_2_ mediates ABA-induced plant stomatal closure and tolerance to heat stress (Zhang et al., 2001; Zhou et al., 2014). Evidence shows that ABA-induced H_2_O_2_ accumulation may be involved in an early expression of diverse antioxidative genes, which contribute to cold stress tolerance (Xuexuan et al., 2010). Interestingly, H_2_O_2_ can work in coordination with nitric oxide, ABA and ethylene in response to cold (Thakur and Nayyar, 2013). Overall, feedback or feed-forward interactions may presumably occur between ABA and H_2_O_2_ in plants response to low temperature stress.

Therefore, further investigation of the relationship between ABA and H_2_O_2_ in the process of SA-induced freezing tolerance in wheat is of interest. Accordingly, the objectives of this study were to (1) understand the effect of exogenous SA on freezing tolerance, (2) clarify the roles of H_2_O_2_ and ABA in SA-induced freezing tolerance, and (3) identify the interaction between H_2_O_2_ and ABA in SA-induced freezing tolerance in wheat.

## Materials and Methods

### Experimental Design

Uniform seeds of winter wheat (*Triticum aestivum* L. cv. Yangmai 16) were selected and surface-sterilized with 2.5% sodium hypochlorite for 10 min and washed several times with sterile distilled water. Seeds were sown into vermiculite and maintained at temperatures of 22°C/18°C (day/night), and 12 h photoperiod with photosynthetically active radiation (PAR) of 400 μmol m^−2^ s^−1^. After 14 days of growth, seedlings were transplanted to a container (40 cm × 35 cm × 18 cm) filled with Hoagland nutrient solution and grown in a controlled environment (with day/night temperature at 22°C/18°C), and 12 h photoperiod under with PAR 400 μmol m^−2^ s^−1^. The solution was renewed daily and aerated over the whole experimental period.

To identify the optimal concentration of exogenous SA, 0, 10, 100, and 1000 μM SA were sprayed on wheat leaves at the four-leaf stage three times, with an interval of 12 h. Freezing stress was applied at 12 h after SA pretreatment, and plants were challenged with freezing at −2°C/400 μmol m^−2^ s^−1^ for 24 h. To investigate the source of endogenous H_2_O_2_ induced by SA, leaves were treated with 5 mM SHAM (an inhibitor of cell wall peroxidase) or 100 μM DPI (an inhibitor of NADPH oxidase) for 8 h before treated the plants with 100 μM SA. To evaluate the crosstalk between H_2_O_2_ and ABA in SA-induced freezing stress, leaves were pretreated with 2 mM DMTU (a H_2_O_2_ and OH• scavenger) or 1 μM Flu (an ABA synthesis inhibitor) for 8 h before the treatment of 100 μM SA. After 12 h of SA treatment, plants were treated with freezing as described above. The last fully expanded leaves were used for the following measurements.

### Chlorophyll Fluorescence Analysis

Chlorophyll fluorescence parameters were detected using an imaging pulse amplitude modulated fluorometer (CF Imager, Technologica, United Kingdom). Seedlings were dark adapted for 30 min at least before taking images and measuring dark-adapted maximum photosystem II quantum yield (*F*v/*F*m) was determined with the entire leaf. The ETR were determined and calculated as actinic irradiance × the quantum yield of photosystem II (ΦPSII) × 0.84 × 0.5, where 0.5 is the supposed proportion of absorbed quanta used by PSII reaction centers, actinic irradiance is 500 μmol m^−2^ s^−1^, and 0.84 is the leaf absorbance for wheat leaves, respectively.

### Electrolyte Leakage Measurement and ABA Quantification

Electrolyte leakage (EL) was tested according to Janda et al. (1999). ABA concentration was determined according to the indirect enzyme-linked immunosorbent assay (ELISA) method described by Liu et al. (2016).

### Antioxidant Enzyme Activity and Malondialdehyde Content

The extractions of antioxidant enzymes were conducted according to Xia et al. (2009) with some modification. Briefly, 0.5 g of leaf samples were ground using a mortar and pestle with 5 mL precooled 50 mM HEPES buffer (pH 7.8) containing 0.2 mM EDTA, 2 mM ascorbic acid, and 2% polyvinylpyrrolidone. The homogenates were then centrifuged at 15,000 *g* for 20 min at 4°C and the supernatants were used for enzymatic activity analysis. SOD (EC 1.15.1.1) activity was determined as described by Patra et al. (1978). The activities of CAT (EC1.11.1.6) and APX (EC 1.11.1.11) were determined by the decrease in A_240_ and A_290_ as described by Xia et al. (2009), respectively. Soluble protein content was tested using the Bradford (1976) method. The content of Malondialdehyde (MDA), an indirect measurement of lipid peroxidation, was determined as described by Xia et al. (2009).

### H_2_O_2_ Detection and Quantification

Cytochemical detection of H_2_O_2_ was described by Zhou et al. (2013) using CeCl_3_ for localization at the subcellular level. The electron-dense CeCl_3_ deposits that are formed in the presence of H_2_O_2_ are visible by transmission electron microscopy. Confocal laser scanning microscopy (CLSM) detection of H_2_O_2_ was followed to the method of Xia et al. (2011) with minor modifications. Intact leaves with the sheath were immersed in 10 mM Tris–HCl (pH 7.4) containing 25 μM 2′,7′-dichlorofluorescein diacetate (H_2_DCFDA) for 6 h at 30°C in a growth chamber without light. After staining, leaves pieces were washed by same buffer and thin sections were cut by hand. The sections were examined with a confocal microscopy using excitation wavelength of 488 nm and emission wavelength of 525 nm (model TCS-SP2 CLSM; Leica Lasertechnik GmbH, Heidelberg, Germany). H_2_O_2_ was measured using assay kits from Jiancheng Bioengineering Institute, China.

### Cell Wall Peroxidase (Prx) Activity

Cell wall Prx (ionically bounded) was extracted as described by Lin and Kao (2001) with NaCl solution. The activity of cell wall Prx was determined by monitoring the changes in A_470_ due to the oxidation of guaiacol as described by Adam et al. (1995).

### Total RNA Extraction and Gene Expression Analysis

Total RNA was isolated from leaves using RNAiso Plus (Takara, Dalian, China) following the manufacturer’s instruction. Two micrograms of total RNA were used as the template for first strand cDNA synthesis according to the manufacturer’s protocol (Sangon, Shanghai, China). The real time quantitative PCR was performed using gene-specific primers and Power SYBR Green PCR Master Mix (Vazyme, Nanjing, China) with a Bio-Rad iCycler iQ5 fluorescence real time PCR system (Bio-Rad, Hercules, CA, United States). The primers for *Prx103*, *Prx108*, *Rboh* (respiratory burst oxidase homolog) *D*, *RbohF*, *NCED* (9-*cis*-epoxycarotenoid dioxygenase) *1*, *NCED2*, *CS* (cold-specific) *120*, *COR* (cold-regulate) *14*, *CBF* (c-repeat binding factor) *3*, *RAB* (response to ABA) *17*, *RAB18*, and *ABI* (ABA insensitive) *5* were listed in Supplementary Table 1. The relative expression levels of genes were calculated using the delta–delta *C*t method (Wang et al., 2018) using *ADP-RF* (ADP-ribosylation factor) as the reference gene as described previously (Paolacci et al., 2009).

### Statistical Analysis

All the data are expressed as the mean ± SD. One-way analysis of variance (ANOVA) was used on the data sets and tested for significant (*P* < 0.05 and *P* < 0.01) treatment differences using Duncan’s multiple range test (SigmaPlot 10.0; Systat Software).

## Results

### Effects of Exogenous SA on Freezing Tolerance of Wheat Plants

**FIGURE 1:**
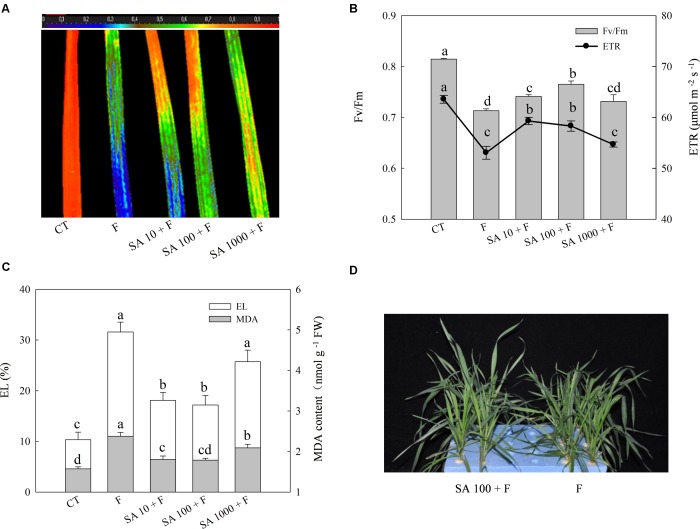
SA pretreatment improves freezing tolerance of wheat plants. **(A)** Image of the maximum PS II quantum yield (*F*v/*F*m). **(B)** The average value of *F*v/*F*m and electron transport rates (ETR). **(C)** Electrolyte leakage (EL) and MDA content. **(D)** The phenotype of wheat plants in response to water and SA (100 μM) under freezing stress. Plants treated with 0, 10, 100, and 1000 μM SA were challenged with freezing stress at –2°C for 1 day, and were denoted as F, SA10 + F, SA100 + F, SA1000 + F, respectively. CT indicates the control (no SA and no freezing stress). The color code depicted on the top of the image **(A)** ranges from 0 (blue) to 1.0 (red). Data are the means ± SD of three replicates. Significant differences at *P* < 0.05 level are denoted by different lowercase letters according to Duncan’s multiple range test.

To evaluate the alleviation effects of exogenous SA on freezing tolerance, we sprayed the foliage of wheat plants with different concentrations of SA and then subjected the plants to freezing stress. The morphology, *F*v/*F*m, ETR, MDA content, and EL were used for evaluation of freezing tolerance. Freezing treatment resulted in prominent decrease of *F*v/*F*m and ETR, while SA pretreatment resulted in less reduction in *F*v/*F*m and ETR of wheat leaves resulted from freezing (**Figures 1A,B**). In addition, SA pretreatment effectively inhibited the increase of MDA content and EL following freezing (**Figure 1C**). We observed that 100 μM SA has a better alleviation effect on *F*v/*F*m reduction than other concentrations. After a few days freezing treatment, severe damage symptoms could be visually observed in water pretreatment plants, while those which were pretreated with 100 μM SA showed less severe symptoms than water pretreatment plants under freezing (**Figure 1D**). Therefore, 100 μM exogenous SA was chosen as the efficient concentration to enhance the capacity of freezing tolerance in wheat plants.

### Exogenous SA Triggers H_2_O_2_ Accumulation in Leaves

**FIGURE 2:**
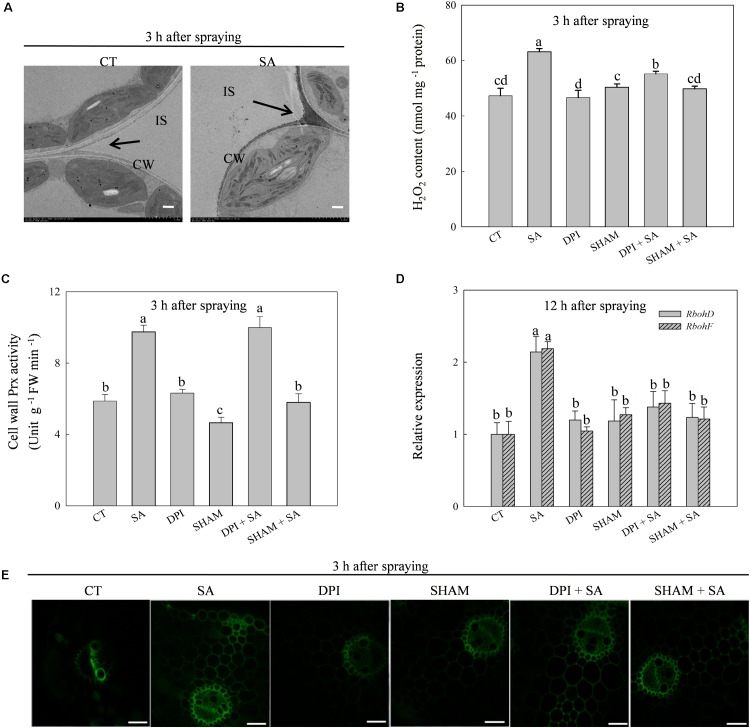
SA regulates H_2_O_2_ production in leaves of wheat plants. **(A)** Cytochemical localization of H_2_O_2_ in wheat leaves with CeCl_3_ staining and transmission electron microscopy. Arrows, CeCl_3_ precipitates; IS, intercellular space; CW, cell wall; Bars, 2 μm. The last fully expanded leaves were harvested at 3 h after water or 100 μM SA treatment. **(B)** Effects of DPI (diphenyleneiodonium, an inhibitor of NADPH oxidase) and SHAM (salicylhydroxamic acid, an inhibitor of cell wall Prx) on SA-induced H_2_O_2_ production. **(C)** Effects of DPI on SA increased the activity of cell wall Prx. **(D)** Effects of SHAM on SA up-regulated expression of *RbohD* and *RbohF*. **(E)** Staining H_2_O_2_ with H_2_DCF-DA and detected by confocal laser scanning microscope system (CLSM). Bars, 200 μm. Plants pretreated with 100 μM DPI or 5 mM SHAM were treated with water or 100 μM SA. The last fully expanded leaves were used for the analysis of H_2_O_2_ content and cell wall Prx activity at 3 h, *Rboh* expression at 12 h after water or 100 μM SA treatment. Data are the means ± SD of three replicates. Significant differences at *P* < 0.05 level are denoted by different lowercase letters according to Duncan’s multiple range test.

The production of H_2_O_2_ was triggered by exogenous SA and reached a peak at 3 h after SA treatment (Supplementary Figures 1A,B). The SA-induced H_2_O_2_ accumulated predominantly on the cell walls in the apoplast (**Figure 2A**). Exogenous SA significantly up-regulated the expression of *Prx103* and *Prx108*, genes encoding cell wall Prx, which catalyzes the production of H_2_O_2_ in the apoplast, as well as the up-regulation of cell wall Prx activity (Supplementary Figure 2). The maximum cell wall Prx activity also appeared at 3 h after SA treatment. Expression of *RbohD* and *RbohF*, genes encoding membrane-linked NADPH oxidase, which also catalyzes the production of H_2_O_2_ in the apoplast, were induced by SA (Supplementary Figure 2). Significant differences in *Rboh*s expression between SA treated and control plants occurred at 12 h after SA treatment, which was much later than the expression of *Prx*s.

These results imply that SA-induced production of H_2_O_2_ originated from the cell wall Prx and then secondarily from membrane-linked NADPH oxidase. To verify this hypothesis, we pretreated leaves with SHAM (an inhibitor of cell wall Prx) or DPI (an inhibitor of NADPH oxidase) before SA treatment. As expected, DPI pretreatment did not affect SA-induced activation of cell wall Prx (**Figure 2C**), while SHAM and DPI pretreatments significantly down-regulated SA-induced expression of *RbohD* and *RbohF* (**Figure 2D**). In addition, DPI pretreatment only partially inhibited SA-induced H_2_O_2_ production, whereas SHAM pretreatment eliminated SA-induced H_2_O_2_ accumulation (**Figure 2B**). The results of H_2_O_2_ measuring by staining with H_2_CDFDA were consistent with the result of chemical assay (**Figure 2E**). These observations suggest that SA-induced extracellular H_2_O_2_ accumulation is initialed by cell wall Prx, and then by activating membrane-linked NADPH oxidase to produce more H_2_O_2_ in the apoplast.

### ABA Participates in SA-Induced H_2_O_2_ Production

**FIGURE 3:**
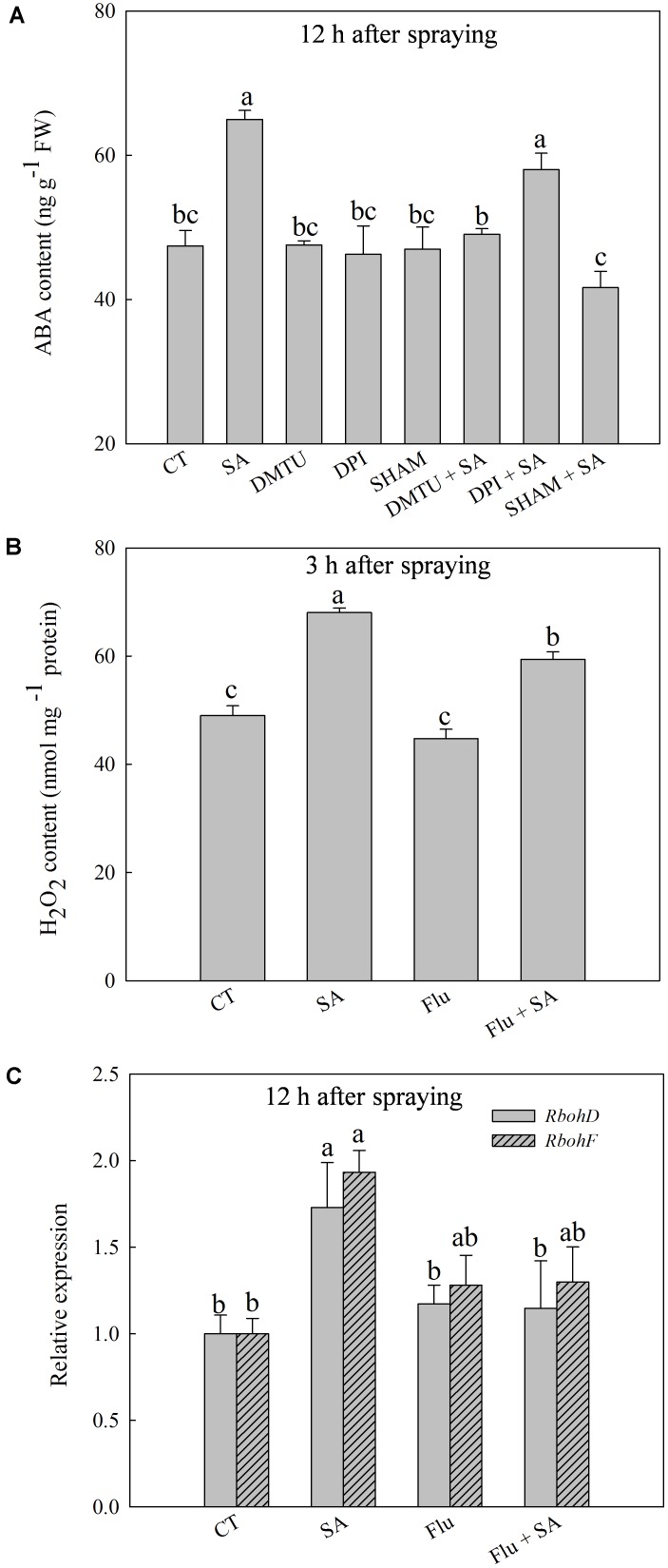
The relationship between SA-induced H_2_O_2_ and ABA. **(A)** Effects of DMTU (dimethylthiourea, a scavenger of H_2_O_2_ and OH•), DPI or SHAM on SA-induced ABA accumulation. **(B)** Effects of Flu (fluorine, an inhibitor of ABA synthesis) on SA-induced H_2_O_2_ accumulation. **(C)** Effects of Flu on SA up-regulated expression of *RbohD* and *RbohF*. Plants pretreated with water, 2 mM DMTU, 100 μM DPI, or 5 mM SHAM were treated with water or 100 μM SA treatment. The last fully expanded leaves were collected for the analysis of H_2_O_2_ content at 3 h, ABA content and *Rboh* expression at 12 h after water or 100 μM SA treatments. Data are the means ± SD of three replicates. Significant differences at *P* < 0.05 level are denoted by different lowercase letters according to Duncan’s multiple range test.

Exogenous SA resulted in a significant increase of endogenous ABA content at 12 h after SA treatment (Supplementary Figure 1C), accompanied with the obviously up-regulated expression of *NCED1* and *NCED2* at 12 h after SA treatment (Supplementary Figure 1D). To explore the interactions of H_2_O_2_ and ABA in SA signal transduction, we analyzed the response of endogenous ABA or H_2_O_2_ to pretreatment with SHAM, DMTU (a H_2_O_2_ and OH• scavenger), DPI, or Flu (an ABA synthesis inhibitor) before SA application. SA-induced ABA accumulation was almost entirely blocked by SHAM or DMTU, while was only slightly inhibited by DPI (**Figure 3A**). Interestingly, Flu also obviously but partially inhibited SA-induced H_2_O_2_ accumulation (**Figure 3B**). In addition, Flu blocked SA-induced expression of *RbohD* and *RbohF* (**Figure 3C**). Basing on these observations, the H_2_O_2_ generated by cell wall Prx appears to act upstream of ABA in response to SA, while ABA could activate NADPH oxidase to release H_2_O_2_ in the apoplast.

### Endogenous H_2_O_2_ and ABA Play Different Roles in SA-Induced Freezing Tolerance

**FIGURE 4:**
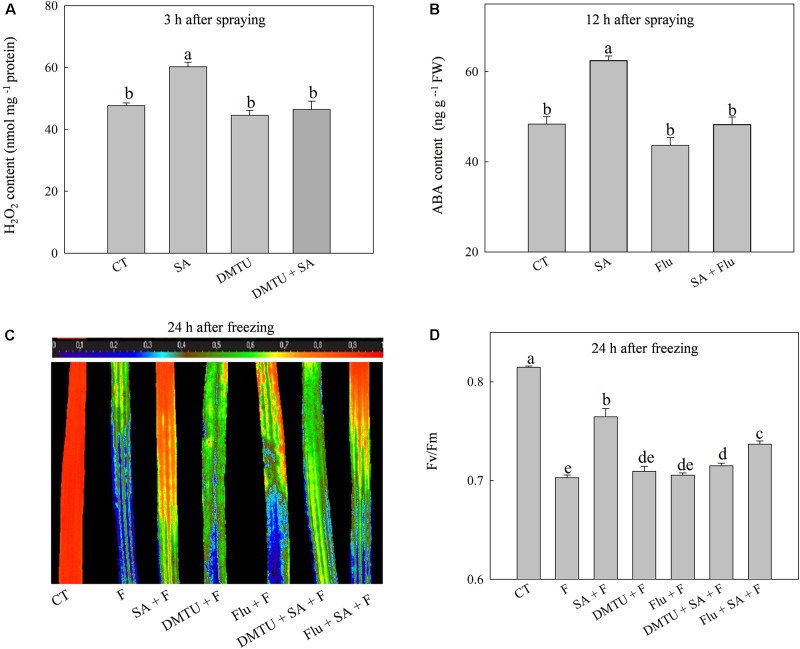
DMTU and Flu offset the SA-induced freezing tolerance. **(A)** Effects of DMTU on SA-induced H_2_O_2_ accumulation at 3 h after water or 100 μM SA treatment. **(B)** Effects of Flu on SA-induced ABA accumulation 12 h after water or 100 μM SA treatment. **(C)** Effects of DMTU or Flu on the images of *F*v/*F*m in SA treated plants under freezing stress. **(D)** Effects of DMTU or Flu on the average value of *F*v/*F*m in SA treated plants under freezing stress. Plants pretreated with water, 2 mM DMTU or 1 μM Flu were treated with water or 100 μM SA. After 12 h, the plants were challenged with freezing stress at –2°C for 1 day. Data are the means ± SD of three replicates. Significant differences at *P* < 0.05 level are denoted by different lowercase letters according to Duncan’s multiple range test.

**FIGURE 5:**
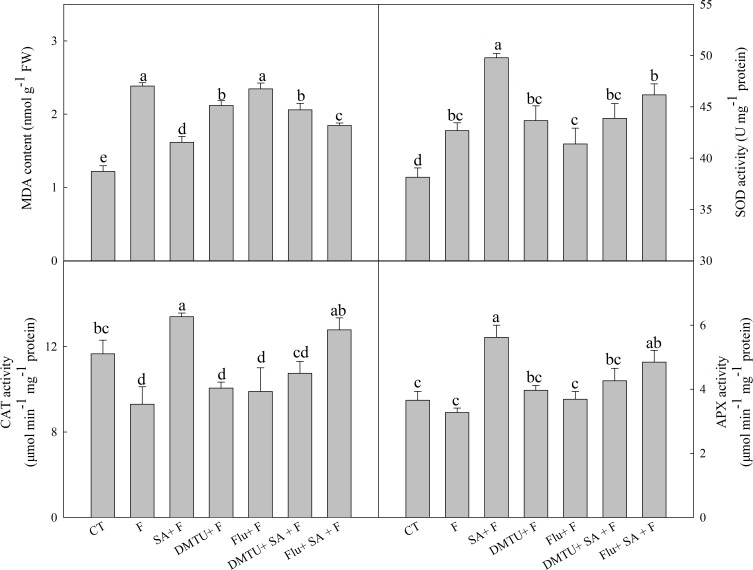
DMTU and Flu reduce the SA-induced increase of antioxidative capacity in wheat under freezing stress. Plants pretreated with water, 2 mM DMTU or 1 μM Flu were treated with water or 100 μM SA. After 12 h, the plants were challenged with freezing stress at –2°C for 1 day. Data are the means ± SD of three replicates. Significant differences at *P* < 0.05 level are denoted by different lowercase letters according to Duncan’s multiple range test.

**FIGURE 6:**
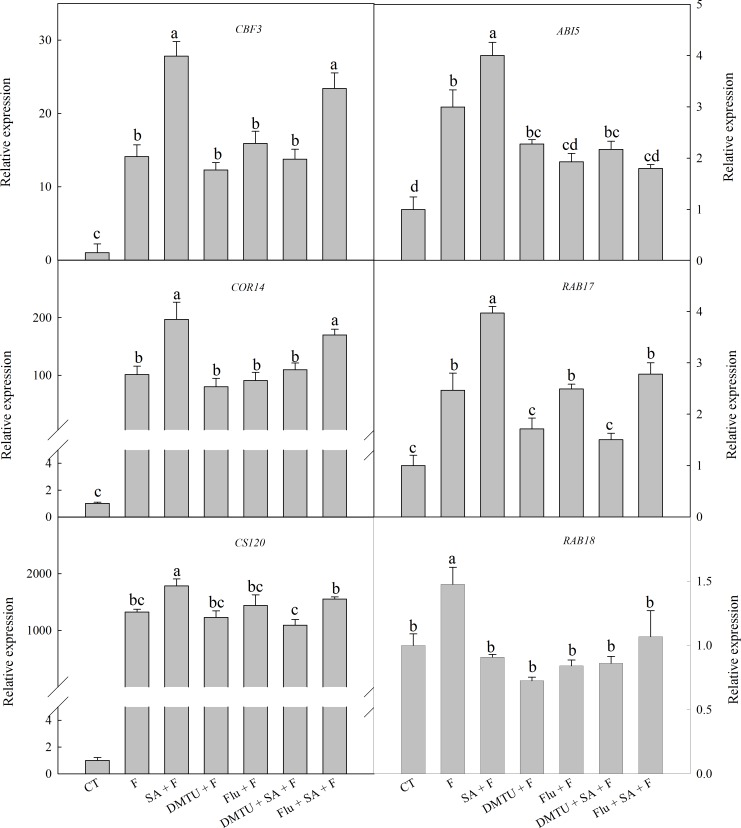
DMTU and Flu down-regulate the SA-induced expression of cold-responsive genes in wheat under freezing stress. Plants pretreated with water, 2 mM DMTU or 1 μM Flu were treated with water or 100 μM SA. After 12 h, the plants were challenged with freezing stress at –2°C for 1 day. Data are the means ± SD of three replicates. Significant differences at *P* < 0.05 level are denoted by different lowercase letters according to Duncan’s multiple range test.

To confirm the functions of H_2_O_2_ and ABA in SA-induced wheat freezing tolerance, we investigated the effects of DMTU or Flu on freezing tolerance in wheat. DMTU and Flu pretreatments completely inhibited SA-induced accumulation of H_2_O_2_ and ABA, respectively (**Figures 4A,B**). However, the protective effect of exogenous SA was almost completely blocked by DMTU while only partially suppressed by Flu when plants were exposed to freezing (**Figures 4C,D**, **5**). Likewise, DMTU substantially eliminated SA-induced activation of SOD, CAT, and APX and the expression of *CBF3, COR14, CS120*, *ABI5*, and *RAB17* in freezing stressed plants (**Figures 5**, **6**). However, Flu only slightly restrained the activation of SOD, CAT, and APX and the expression of *CBF3, COR14*, and *CS120*, but blocked the expression of *ABI5* and *RAB17* induced by SA in freezing stressed plants (**Figures 5**, **6**). Exogenous application of ABA naturally alleviated the inhibition of PS II-induced by freezing while H_2_O_2_ showed no sign of this mitigative effect (Supplementary Figure 3). Collectively, these results demonstrate that both H_2_O_2_ and ABA are involved in SA-induced freezing tolerance in wheat plants, and H_2_O_2_ may play an upstream role relative to ABA.

## Discussion

### SA Induces Freezing Stress Tolerance by Up-Regulating Antioxidant Capacity and Cold-Responsive Genes Expression in Wheat

Numerous researches have indicated that exogenous SA can enhance plant tolerance to freezing stress embodying maintenance of photosynthesis capacity, reduction of electrolyte leakage and MDA content (Janda et al., 1999; Fei et al., 2016). However, SA-induced stress tolerance could be plant growth stages, species and SA dose dependent (Fei et al., 2016). In this study, exogenous SA (at any concentrations of 10, 100, and 1000 μM) induced freezing tolerance by increasing *F*v/*F*m, decreasing electrolyte leakage and MDA content as compared to freezing only treatment in wheat (**Figure 1**), and 100 μM was the optimum concentration of those tested.

The antioxidant systems play crucial roles in reactive oxygen species (ROS) scavenging and show a positive correlation with plant freezing tolerance (Kang et al., 2013). Several studies illustrated that SA could enhance antioxidant capacity in various plant species, such as maize, rice, and cucumber (Kang and Saltveit, 2002). In this study, exogenous SA (100 μM) notably increased the activities of SOD, CAT, and APX, which was consistent with the lower MDA content in comparison with no pretreatment under freezing stress (**Figure 5**). The above results confirm that exogenous SA effectively relieves cell membrane damage induced by freezing at the level of antioxidant defense in wheat plants.

The accumulation of COR proteins are one of the critical mechanisms of the plant to cope with freezing stress (Kobayashi et al., 2008). COR14, CS120, RAB17, and RAB18 are typical of COR proteins (Ganeshan et al., 2008; Kobayashi et al., 2008). Induction of *Cor* genes by low temperature stress is regulated by CBF and ABA-responsive element binding protein (AREB) (Kobayashi et al., 2008). The activation of *CBF* genes expression is also ABA-dependent and ABA-independent (Zhou et al., 2011). Among the six selected genes in this study, the expression of *CBF3, COR14*, and *CS120* are ABA-independent, while the expression of *ABI5* (show highly homologous to AREB2), *RAB17*, and *RAB18* are ABA-dependent (Houde et al., 1992; Tsvetanov et al., 2000; Kobayashi et al., 2008). In the present study, freezing dramatically up-regulated the expression of these cold-responsive genes, especially *CBF3, COR14*, and *CS120*, which were well accordance in with previous finding that CBF-COR pathway have a crucial role in constitutive freezing tolerance in plants (Chinnusamy et al., 2007). However, the effects of SA on these cold-responsive genes expression is still controversial. Miura and Ohta (2010) found that SA-accumulating mutant *siz1* exhibits sensitivity to freezing, which is related to the down-regulated expression of *CBF3*. Nevertheless, PAC (paclobutrazol), a SA synthesis inhibitor, suppress the expression of *CBF, COR47*, and *RAB18* in chilling stressed cucumber seedlings, and the decrement in the gene expression can be restored by exogenous SA (Dong et al., 2014). Most recently, Ding et al. (2016) found that exogenous SA up-regulated the expression of *CBF1*, which protect tomato fruit from chilling injury. In the present study, SA pretreatment significantly up-regulated the expression of *CBF3*, *COR14*, *CS120*, *ABI5*, and *RAB17* (**Figure 6**), suggesting that SA enhanced wheat freezing tolerance by up-regulating the expression of both the ABA dependent and independent cold-responsive genes.

### SA Triggers Extracellular H_2_O_2_ Production First Mediated by Cell Wall Prx and Further Magnified by the Membrane Linked NADPH Oxidase

There are three major pathways for the production of endogenous H_2_O_2_ under SA-induced abiotic and biotic stress tolerance. First, SA binds with CAT to suppress its activity, which leads to H_2_O_2_ accumulation (Chen et al., 1993; Kang et al., 2003). Second, SA activates membrane-linked NADPH oxidase mediating H_2_O_2_ production, which occurs in the apoplast (Kalachova et al., 2013). Third, SA induces extracellular H_2_O_2_ production via the SHAM-sensitive cell wall Prx (Mori et al., 2001; Khokon et al., 2011). However, the pathway of SA-induced endogenous H_2_O_2_ production in wheat remains unclear. In this study, SA-induced endogenous H_2_O_2_ accumulation was located on cell walls in the apoplast (**Figure 2A**). SA significantly enhanced cell wall Prx activity and up-regulated the expression of cell wall Prx and membrane-linked NADPH oxidase encoding genes (Supplementary Figure 2). Pretreated with SHAM (an inhibitor of cell wall Prx) and DPI (an inhibitor of NADPH oxidase) markedly reduced SA-induced H_2_O_2_ accumulation (**Figures 2B,E**). The above results suggest that both cell wall Prx and membrane-linked NADPH oxidase mediate SA-induced extracellular H_2_O_2_ accumulation in wheat. It should be noted that SHAM is also an inhibitor of the alternative oxidase (AOX), which is the terminal oxidase in the cyanide-resistant respiration pathway (Wei et al., 2015). The AOX pathway have an important role in decreasing the mitochondrial ROS level by reducing oxygen to water without conservation of energy in the form of ATP (Sugie et al., 2006). Here, SHAM treatment slightly increased the content of H_2_O_2_ as compared to water treatment (CT), indicating that SHAM may suppress the activity of AOX to induce ROS production (**Figure 2B**). However, pretreated with SHAM significantly decreased SA-induced H_2_O_2_ accumulation (**Figure 2B**). These findings reveal that SHAM reduced SA-induced H_2_O_2_ production by inhibiting cell wall Prx activity.

Recently, O’Brien et al. (2012) proposed that the cell wall Prx derived H_2_O_2_ production is the main source of ROS burst with the complementary sources of the NADPH oxidase-derived ROS production etc. In the present study, SA-induced H_2_O_2_ accumulation was completely blocked by SHAM and partially suppressed by DPI (**Figures 2B,E**). Pretreatment with DPI did not affect SA-induced cell wall Prx activity (**Figure 2C**), whereas SHAM completely blocked SA-induced expression of *RbohD* and *RbohF* (**Figure 2D**), indicating that SA-induced extracellular H_2_O_2_ production is first activated by cell wall Prx, and this original H_2_O_2_ further acted as a signal to activate NADPH oxidase to exaggerate the production of H_2_O_2_ in the apoplast.

### The Crosstalk of Endogenous H_2_O_2_ and ABA in Response to SA

Several lines of evidences show that ABA induces apoplastic H_2_O_2_ production via NADPH oxidase to result in plant tolerance to heat and oxidation stress (Zhou et al., 2014). Exogenous brassinosteroids (BRs) induced ABA synthesis in wild type plants, but failed in the *RBOH1*-silenced mutants, demonstrating that H_2_O_2_ is pivotal in BRs-induced ABA biosynthesis (Zhou et al., 2014). Previous findings hint that there is a cross talk between H_2_O_2_ and ABA signals in response to external stresses and hormones. Here, pretreatment with SHAM and DMTU eliminated SA-induced accumulation of ABA (**Figure 3A**), indicating that H_2_O_2_ originated from the cell wall Prx can serve as a signal to mediate SA-induced ABA accumulation. However, DPI pretreatment had very little impact on SA-induced ABA synthesis (**Figure 3A**). Furthermore, inhibiting ABA synthesis by Flu markedly down-regulated SA-induced expression of *RbohF* and *RbohD* and the accumulation of H_2_O_2_ (**Figures 3B,C**). Thus, ABA signal can mediate SA-induced H_2_O_2_ originated from the membrane-linked NADPH oxidase. These results suggest that SA-induced ABA accumulation is dependent on the burst of H_2_O_2_ generated by cell wall Prx, and the induced ABA causes a further increase in H_2_O_2_ production in the apoplast by activating NADPH oxidase.

### H_2_O_2_ and ABA Mediate SA-Induced Freezing Tolerance

The relationship between SA and H_2_O_2_ in plants response to biotic and abiotic stress is still not fully understood. An increasing body of evidence supports that SA can work in concert with H_2_O_2_ to mediate plant senescence, chilling tolerance and SAR (Kang et al., 2003; Vlot et al., 2009; Yoshimoto et al., 2009), whereas the others reported that SA-induced abiotic or biotic stress tolerance are H_2_O_2_ independent (Bi et al., 1995; Mora-Herrera et al., 2005). In this study, SA-induced freezing tolerance was accompanied with elevated H_2_O_2_ content (**Figure 2B** and Supplementary Figures 1A,B), and scavenging H_2_O_2_ by DMTU (DMTU + SA + F) eliminated SA-induced freezing tolerance as demonstrated by insignificant differences in *F*v/*F*m and activities of the antioxidant enzymes (**Figures 4A,C,D**, **5**) and expression of cold-responsive genes as compared to SA pretreatment alone (SA + F, **Figure 6**). These results highlight the crucial role of H_2_O_2_ in SA-induced freezing tolerance in wheat plants. Our results consistent with previous studies reported that apoplast ROS has an important role in mediating long-distance systemic signaling in plants in response to diverse environmental stimuli (Miller et al., 2009), and apoplast H_2_O_2_ can act as signal molecules in the activation of stress responses and induction of cold tolerance in cucumber (Xia et al., 2009) and freezing tolerance in wheat (Si et al., 2017). Exogenous H_2_O_2_ also has been shown to induce plants cold tolerance by up-regulating the activities of antioxidant enzymes and expression of cold responsive genes (Xia et al., 2009; Li et al., 2017). However, the findings of our study showed that pretreated with exogenous H_2_O_2_ had no alleviative effect on the inhibition of PS II caused by freezing stress. The contradiction between the previous and our results may be due to the different concentrations of applied H_2_O_2_ and temperatures of setting used in experiments (Xu et al., 2011; Si et al., 2017). Together, we suggested that exogenous SA-induced freezing tolerance in wheat is mediated by apoplast H_2_O_2_, which derives first from the cell wall Prx and then from the NADPH oxidase.

Salicylic acid-induced plant stress tolerance has been considered to be mediated by endogenous ABA signal (Szepesia et al., 2009; Jesus et al., 2015). Exogenous SA induces ABA accumulation by up-regulating of ABA biosynthesis related genes (Horvath et al., 2015). Recently, Shakirova et al. (2016) found that endogenous ABA plays a key role in SA-induced cadmium stress tolerance in wheat. In this study, exogenous SA treatment resulted in significantly higher ABA content (**Figure 3A** and Supplementary Figure 1C), by up-regulating the expression of *NCED1* and *NCED2*, key genes involving in ABA synthesis (Supplementary Figure 1D). Moreover, the ABA biosynthesis inhibitor Flu (Flu + SA + F) prominently reduced the alleviation effect of SA on freezing tolerance as indicted by the less significant difference in *F*v/*F*m, antioxidant enzymes activities (**Figures 4B–D**, **5**) and the insignificant difference in the expression of ABA-responsive genes, such as *ABI5* and *RAB17* (**Figure 6**), as compared with SA + F. In addition, pretreatment with exogenous ABA showed lower inhibition of PS II under freezing than no pretreatment (Supplementary Figure 3), which in accordance with previous findings that exogenous ABA can effectively enhanced plants tolerance to freezing (Janowiak et al., 2002; Huang et al., 2017). Collectively, these results supported that the involvement of ABA in SA-induced freezing tolerance in wheat plants, and H_2_O_2_ may play an upstream role relative to ABA.

## Conclusion

**FIGURE 7:**
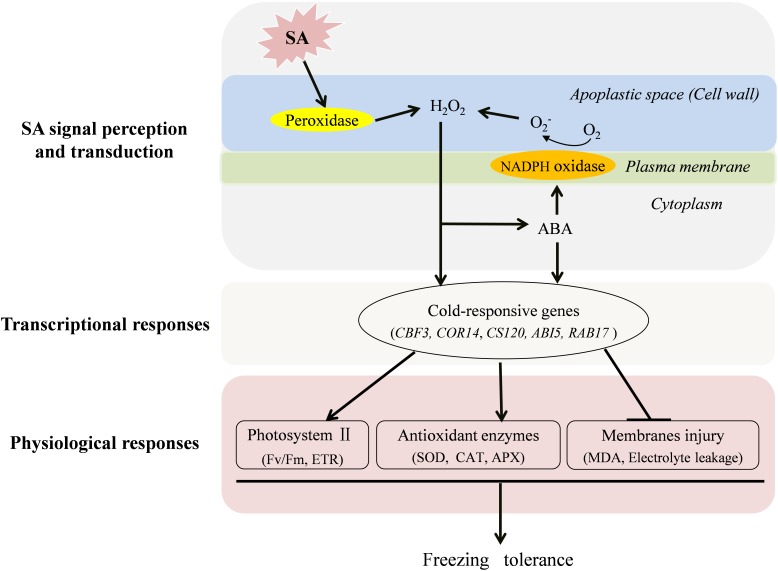
A conclusive model for the induction of freezing tolerance by exogenous SA in wheat plants. SA activates cell wall Prx to produce H_2_O_2_ in the apoplast, which functions as a signal molecule to induce the increase of antioxidative capacity, to trigger the expression of cold-responsive genes and ABA synthesis. The increased ABA further activates NADPH oxidase, which could induce more production H_2_O_2_ in the apoplast. In addition, ABA signal also plays a role in SA-induced antioxidant capacity, and expression of cold-responsive genes in wheat plants under freezing stress.

As concluded in **Figure 7**, both endogenous H_2_O_2_ and ABA play important roles in SA-induced freezing tolerance. Following the perception of a SA signal, cell wall Prx is activated to produce H_2_O_2_ in the apoplast. The increased H_2_O_2_ triggers ABA production, which in turn give rise to a further accumulation of H_2_O_2_ by activating NADPH oxidase leading to prolonged stress tolerance. Therefore, H_2_O_2_ and ABA may form a positive feedback loop to mediate SA-induced freezing tolerance in wheat plants. Finally, the increased H_2_O_2_ and ABA separately trigger their defense pathways to up-regulate expression of cold-responsive genes and activities of antioxidant enzymes to reduce the electrolyte leakage and cell membrane peroxidation and ultimately improve photosynthesis capacity (*F*v/*F*m and ETR) of wheat under freezing stress.

## Author Contributions

WW, XW, and DJ conceived and designed the research. WW and MH conducted the experiments. JC, QZ, TD, and WC guided the experiments. WW wrote the manuscript. All authors read and approved the manuscript.

## Conflict of Interest Statement

The authors declare that the research was conducted in the absence of any commercial or financial relationships that could be construed as a potential conflict of interest.

## Abbreviations

ABA: abscisic acid
APX: ascorbate peroxidase
CAT: catalase
DMTU: dimethylthiourea
DPI: diphenyleneiodonium
ETR: electron transport rates
Flu: fluridone
*F*v/*F*m: maximum PS II quantum yield
H_2_O_2_: hydrogen peroxide
MDA: malondialdehyde
SA: salicylic acid
SHAM: salicylhydroxamic acid
SOD: superoxide dismutase
