# Supplementary material for: Hydrogen Peroxide and Abscisic Acid Mediate Salicylic Acid-Induced Freezing Tolerance in Wheat
Source: Front Plant Sci. 2018 Aug 3;9:1137. doi: 10.3389/fpls.2018.01137 (PMC6085453; doi:10.3389/fpls.2018.01137)
Supplement: Supplementary file 1 [file Table_1.doc]

***Supplementary Material***

**Hydrogen peroxide and abscisic acid mediate salicylic acid-induced freezing tolerance in wheat**

**Weiling Wang, Xiao Wang*, Mei Huang, Jian Cai, Qin Zhou, Tingbo Dai, Weixing Cao, Dong Jiang***

*** Correspondence:** Xiao Wang: [xiaowang@njau.edu.cn](mailto:xiaowang@njau.edu.cn) ; Dong Jiang: [jiangd@njau.edu.cn](mailto:jiangd@njau.edu.cn)


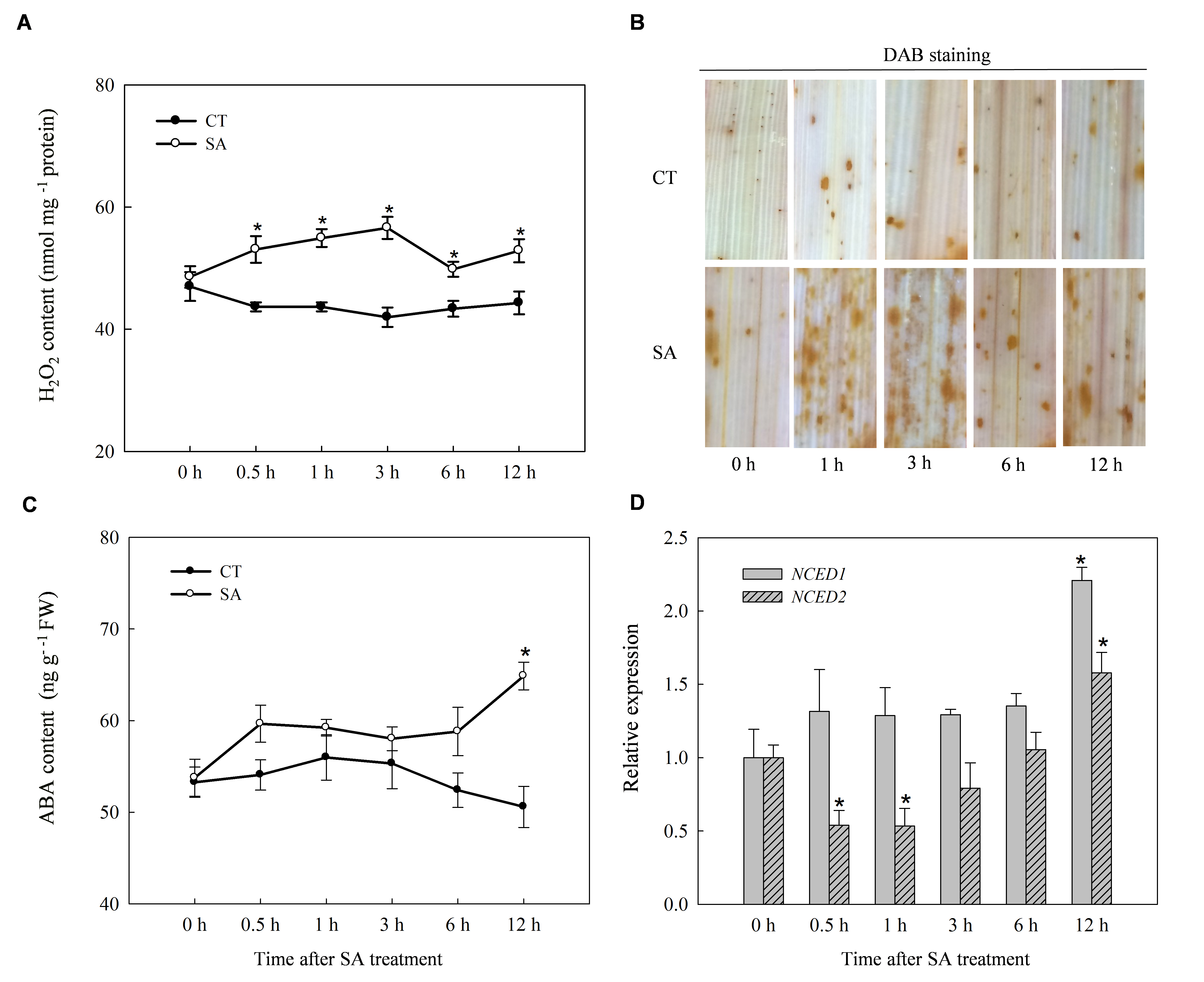
**1 Supplementary** **Figures and Tables**

**1.1 Supplementary Figures**

**Supplementary Figure 1** Kinetic changes in contents of H_2_O_2_ and ABA, and expression levels of *NCED1* and *NCED2* in response to SA in wheat plants. **(A)** H_2_O_2_ content. **(B)** Images of DAB (3,3′-diaminobenzidine) stained leaves showing localized H_2_O_2_ accumulation as dark-brown spots. The DAB staining of H_2_O_2_ production was conducted according to Xia et al. (2009). **(C)** ABA content. **(D)** Expression levels of *NCED1* and *NCED2*. Plants were treated with water or 100 μM SA. The last fully expanded leaves were used for the analysis. Data are the means ± SD of three replicates. * and ** indicated significance differences at *P* < 0.05 and *P* < 0.01, respectively.


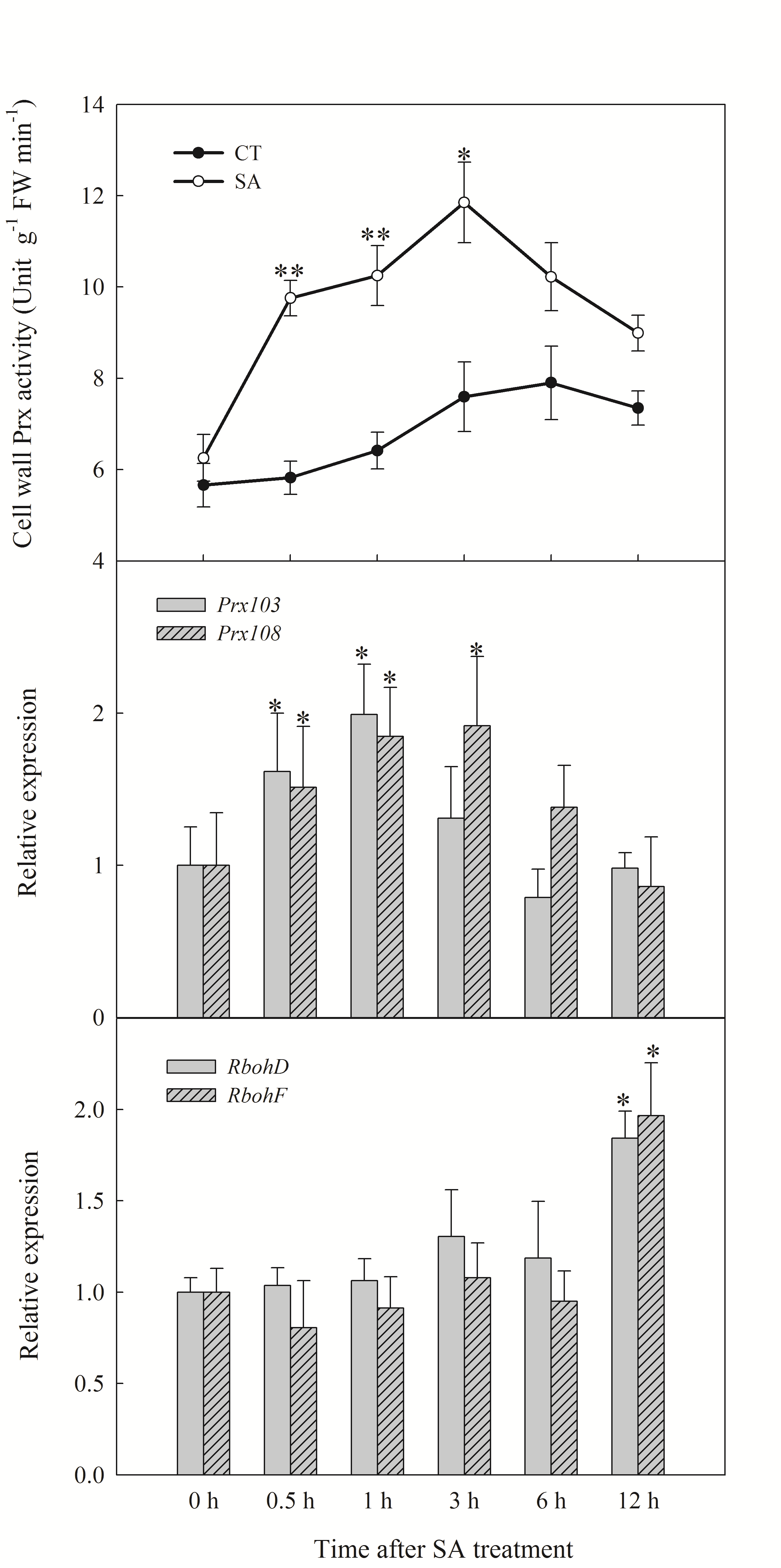
**Supplementary Figure 2** Kinetic changes in activity of cell wall Prx, and expression levels of *Prx103*, *Prx108*, *RbohD* and *RbohF* in response to SA in wheat plants. Plants were treated with water or 100 μM SA. The last fully expanded leaves were used for the analysis. Data are the means ± SD of three replicates. * and ** indicated significance differences at *P* < 0.05 and *P* < 0.01, respectively.


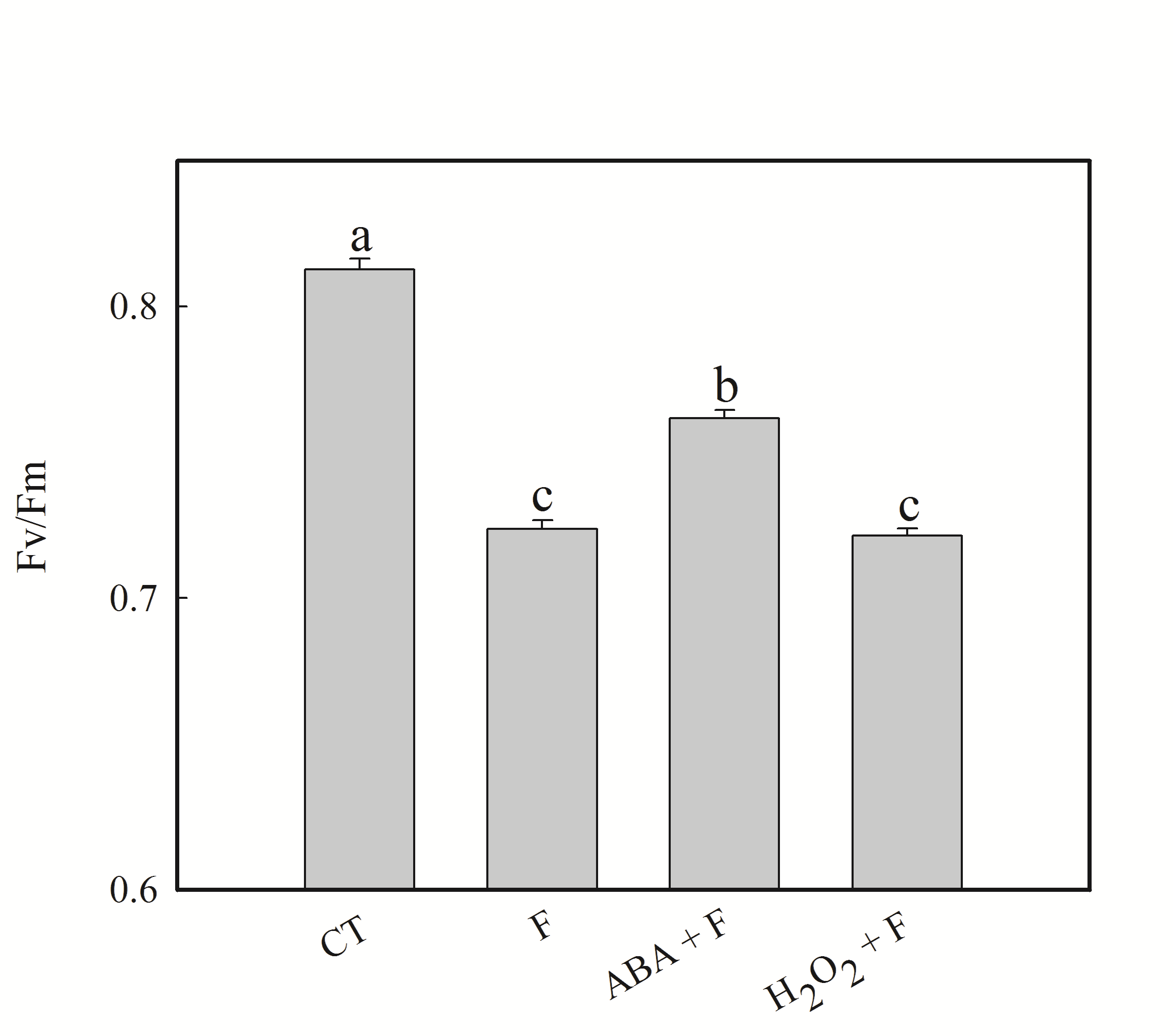
**Supplementary Figure 3** Effects of abscisic acid (ABA) and hydrogen peroxide (H_2_O_2_) on freezing tolerance of wheat plants. Plants treated with water, 10 μM ABA and 200 μM H_2_O_2_ were challenged with freezing stress at -2°C for 1 d, and were denoted as F, ABA + F, H_2_O_2_ + F, respectively. CT indicates the blank control. Data are the means ± SD of three replicates. Significant differences at P < 0.05 level are denoted by different lowercase letters according to Duncan’s multiple range test.

**1.2 Supplementary Tables**

**Supplementary Table 1** Sequences of primers used for real-time PCR amplification and the resulting product size.

| Gene | Forward（F）and reverse（R）primers | Primer sequence 5**’** to 3**’** | Amplicon size (bp) |
| --- | --- | --- | --- |
| *Ta CBF3* | F  R | AATCGTCGTCTGAGTCTGACAGTG  TTCCGGGAACAAGTCAAGCCT | 123 |
| *Ta COR14* | F  R | CGACCACCAGACCCAGACC  CGAGCGGCGAGGAAACAC | 124 |
| *Ta CS120* | F  R | TTCACGGACAACAGTGTG  CTGCGTCTGTCTCTTGGATAAG | 108 |
| *Ta ABI5* | F  R | CACCCTCAGCGCCAAGAC  CTCCCATACCAACTGCCCTC | 142 |
| *Ta RAB17* | F  R | GACGCCAAGGACGCTGTCATG  CAAGCAAGCGCAGACGCG | 102 |
| *Ta RAB18* | F  R | TGAGAGAGAAGAGCAGAGAAGAC  CCGGTCTTCTCCTGCACA | 198 |
| *Ta Prx108* | F  R | TAACCTCACCCAATCCTTCG  CGAAGTTGTTGACCGTGTTG | 142 |
| *Ta Prx103* | F  R | GCATACTAGCCAGCACGACA  ACACGGTTTCAAGAGCTGGT | 85 |
| *Ta RbohD* | F  R | ACCACCAGACCAGACCAGAC  TGGTTGGATAGGAGGCGTAG | 70 |
| *Ta RbohF* | F  R | TGGCACCCCTTCTCAATTAC  CTCTCGTGTCCAGTCACCAA | 84 |
| *Ta NCED1* | F  R | TTCAACGACACGGACGACCAC  TACCTCGTCTTCCGGCCCAG | 163 |
| *Ta NCED2* | F  R | TACATTCTCACCTTCGTTCACGACG  TGCCGTGGAAGCCGTACG | 127 |
| *Ta ADP-RF* | F  R | GCTCTCCAACAACATTGCCAAC  GCTTCTGCCTGTCACATACGC | 165 |

**REFERENCES**

Xia, X.-J., Wang, Y.-J., Zhou, Y.-H., YuanTao, Mao, W.-H., Shi, K., et al. (2009). Reactive oxygen species are involved in brassinosteroid-induced stress tolerance in cucumber. *Plant Physiol.* 150, 801-814.
